# Acute mesenteric ischemia in a newborn with COVID-19: A case report

**DOI:** 10.1016/j.ijscr.2022.107548

**Published:** 2022-08-24

**Authors:** Gonca Gerçel, Ali İhsan Anadolulu

**Affiliations:** a Şanlıurfa Training and Research Hospital Clinic of Pediatric Surgery, Şanlıurfa, Turkey; b Mehmet Akif İnan Training and Research Hospital Clinic of Pediatric Surgery, Şanlıurfa, Turkey

**Keywords:** COVID-19, Newborn, Acute mesenteric ischemia, Case report

## Abstract

**Introduction and importance:**

We aimed to report a case of acute mesenteric ischemia in a newborn with COVID-19.

**Case presentation:**

A 1-day-old male baby, with a birth weight of 2050 g, delivered by spontaneous vaginal delivery at 34 weeks of gestation from a 32-year-old COVID-19 infected mother in her third pregnancy, was taken to the newborn intensive care unit. On physical examination, the patient was alive and active. The abdomen was soft. Laboratory values of the patient were within the normal range. Echocardiography and abdominal ultrasonography were normal. COVID-19 PCR test drawn at 48 h of age was positive. On the postnatal 4th day, the patient suddenly had tachycardia and abdominal tension. Free air in the abdomen was detected on direct abdominal X-ray. The patient was taken to surgery urgently. On laparotomy, brownish ascites and necrotic small bowel and colon starting from 20 cm of ligamentum of Treitz to the middle part of the transverse colon were seen. Jejunostomy was constructed at area that 50 cm distal to Treitz with a relatively better appearance (with circulatory disorder but not full-thickness necrosis) and transverse colon mucous fistula without primary anastomosis. The patient died one day after surgery due to cardiorespiratory arrest and multiorgan failure.

**Conclusions:**

Although most of the reported symptoms of the COVID-19 are related to the respiratory system, there is concern that the occurrence of serious and life-threatening manifestations such as mesenteric ischemia in the gastrointestinal tract may be overlooked in also neonatal period.

## Highlights

• COVID-19, nowadays known as severe acute respiratory syndrome
• COVID-19 infection occasionally involves atypical presentations, such as gastrointestinal manifestation and thromboembolic complications
• We report a case of acute mesenteric ischemia (AMI) in a newborn with COVID-19

## 1 Introduction

In December 2019, the novel coronavirus disease (COVID-19), nowadays known as severe acute respiratory syndrome coronavirus 2 (SARS-CoV-2), was first identified in Wuhan city of China, and quickly spread around the world [1].

The elderly population with underlying diseases are more susceptible to the virus, but the disease can be seen in the childhood and neonatal periods, too. Children are reported to have fewer symptoms and a lower case-death rate, however, a small proportion developed severe disease requiring intensive care unit admission and prolonged ventilation and fatal outcome [2], [3], [4].

Although COVID-19 causes mainly the respiratory complications, it has now become clear that COVID-19 infection occasionally involves atypical presentations, such as gastrointestinal manifestation and thromboembolic complications [5], [6].

To date, limited cases of these lethal patient subgroup have been reported in the world especially in pediatric age group. Herein, we report a case of acute mesenteric ischemia (AMI) in a newborn with COVID-19. The work has been reported in line with the SCARE criteria [7].

## 2 Case presentation

A 32-year-old G3P3 mother presented to the hospital in preterm labor at 34 3/7 weeks gestation. Upon admission to the labor and delivery unit, the mother tested positive for SARS-CoV-2 by routine nasopharyngeal polymerase chain reaction (PCR) screening. The mother had no known additional disease and no history of drug use during pregnancy. A late preterm male infant weighing 2050 g, was born by spontaneous vaginal delivery with APGAR scores of 8 and 10 at 1 and 5 min, and pulse, blood pressure and temperature within normal ranges. The baby was transferred to the intensive care unit because he was premature and had mild respiratory distress. Non-invasive respiratory support with high-flow nasal oxygen was started. The neonate was empirically started on intravenous ampicillin and gentamicin infusions, per suspected neonatal sepsis protocol. On physical examination, the patient was alive and active. The abdomen was soft. Neurological examination was normal. Septic findings were not observed in the patient. Laboratory values of the patient were within the normal range. An initial complete blood count revealed no anemia or leukocytosis, with hemoglobin of 15.1 g/dL, platelet clumping with a normal count (222,000/μL) and white blood count of 14,300/μL. The first SARS-CoV-2 PCR test, drawn per Centers for Disease Control and Prevention (CDC) guidelines at 24 h of age, was negative.

The patient's respiratory distress improved on the first postnatal day. He was breathing room air without tachypnea or increased work of breathing and was started on oral feeds of expressed breast milk and supplemented with formula when breast milk was not available. Echocardiography of the patient revealed patent foramen ovale. Abdominal ultrasonography (USG), transfontanelle USG and portal venous doppler USG were normal on the postnatal 2nd day. The second SARS-CoV-2 PCR test drawn at 48 h of age was positive.

**Fig. 1:**
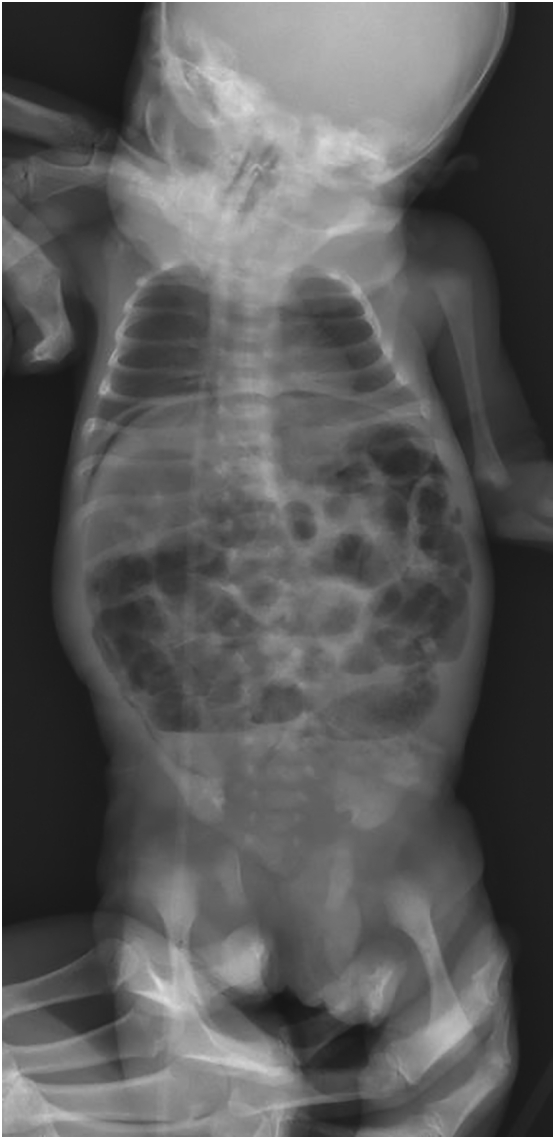
Free intraabdominal air on direct abdominal X-ray.

On the postnatal 4th day, the patient suddenly had tachycardia and abdominal tension. Routine blood tests were repeated and an abdominal X-ray was taken. Routine blood tests, infection markers and coagulation parameters are within normal limits. Free air in the abdomen was detected on direct abdominal X-ray (Fig. 1). The patient was taken to surgery urgently. Surgery was performed by consultant pediatric surgeon.

**Fig. 2:**
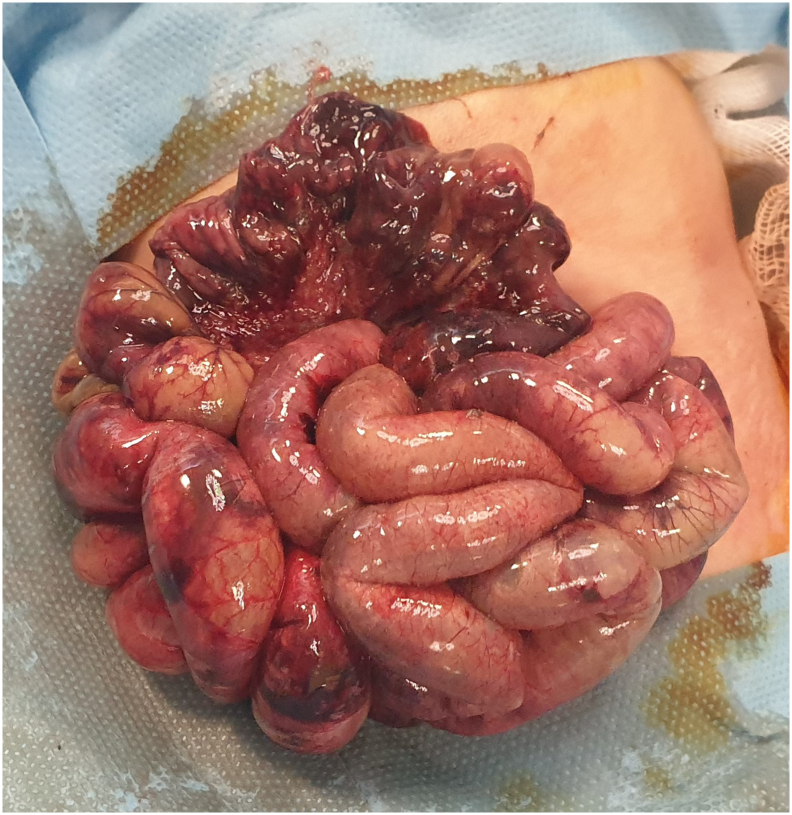
The view of necrotic small intestine.

Upon exploration, brownish ascites and necrotic small bowel and colon starting from 20 cm of ligamentum of Treitz to the middle part of the transverse colon were seen (Fig. 2). We constructed jejunostomy at area that 50 cm distal to Treitz with a relatively better appearance (with circulatory disorder but not full-thickness necrosis) and transverse colon mucous fistula without primary anastomosis. Unfortunately, the patient died one day after surgery due to cardiorespiratory arrest and multiorgan failure.

## 3 Discussion

COVID-19 has been known a respiratory disease, mainly pneumonia. In addition to affecting the respiratory tract and lungs, the disease can cause thrombogenic ischemia in various parts of the body, including the gastrointestinal tract [5], [6]. However, the mechanism of ischemic and tromboembolic complications in patients with COVID-19 are still unclear. Emerging evidence suggests that it could be associated with endothelitis by difuse endothelial damage and infiltration of infammatory cells, and a systemic hypercoagulable state caused by hyperinfammation and hypercytokinemia [8], [9].

Although there are relatively more publications on COVID-19 and ischemic complications in adults, there are very few reports on neonatal patients. Case series of COVID-19 in neonates have identified a variety of clinical presentations from asymptomatic to severe disease, most often presenting with respiratory distress [10].

Necrotizing enterocolitis (NEC) is a significant cause of morbidity and mortality among infants. Although NEC could be observed in neonates of all sizes and gestational age, it is much more commonly seen in infants born less than 1500 g in weight and less than 28 weeks in gestation [11]. The pathophysiology of NEC is multifactorial, including intestinal immaturity, microbial dysbiosis, compromised intestinal epithelial barrier, undeveloped immune defense, altered vascular development and tone, hypoxia, formula feedings and antibiotic exposure [12]. It has been reported in the publications that the mean age of onset is around the 20th day [12], [13].

The patient we present here was born at 34 weeks gestation, over 2000 g at the time of birth, and there was a sudden onset of general condition disorder on the postnatal 4th day which is much earlier than frequently seen with NEC. In the surgical exploration, it was determined that the intestinal sections fed by SMA were almost completely necrotic. Although we do not have any imaging related to this, it was thought that this situation may be due to an ischemic and/or thromboembolic event related to COVID-19. To the best of our knowledge, this is the first report presenting a newborn with COVID-19 who underwent surgery for acute mesenteric ischemia. This may indicate that COVİD-19 antibodies may have caused a nonclassical NEC symptoms in the early neonatal period.

## 4 Conclusion

Although most of the reported symptoms of the COVID-19 are related to the respiratory system, there is concern that the occurrence of serious and life-threatening manifestations such as mesenteric ischemia in the gastrointestinal tract may be overlooked in also neonatal period.

## Sources of funding

The authors declared that this study has received no financial support.

## Ethics approval and consent to participate

Ethics committee approval was not obtained because this was a retrospective case report study and there is no legal obligation for case report studies in our country. Written informed consent was obtained from patient's family prior to surgery included in this study.

## Consent for publication

Written informed consent was obtained from the patient for publication of this case report and accompanying images. A copy of the written consent is available for review by the Editor-in-Chief of this journal on request.

## Availibility of data and materials

All patient data accessible from the Republic of Turkey Ministry of Health system.

## Registration of research studies

Note applicable.

## Guatantor

Dr. Gonca Gerçel

## Provenance and peer review

Not commissioned, externally peer-reviewed.

## CRediT authorship contribution statement

Study Concept and Design: GG, AİA. Data collection: GG, AİA. Analysis and interpretation of data: GG, AİA. Drafting of the manuscript: GG, AİA. All authors read and approved the final manuscript.

## Declaration of competing interest

No conflict of interest was declared by the authors.
